# Glu20Ter Variant in *PLEC* 1f Isoform Causes Limb-Girdle Muscle Dystrophy with Lung Injury

**DOI:** 10.3389/fneur.2017.00367

**Published:** 2017-07-31

**Authors:** Roman V. Deev, Sergei N. Bardakov, Mikhail O. Mavlikeev, Ivan A. Yakovlev, Zoya R. Umakhanova, Patimat G. Akhmedova, Raisat M. Magomedova, Irina A. Chekmaryeva, Gimat D. Dalgatov, Artur A. Isaev

**Affiliations:** ^1^Human Stem Cells Institute, Moscow, Russia; ^2^Ryazan State Medical University, Ryazan, Russia; ^3^Department of Neurology, S.M. Kirov Military Medical Academy, St. Petersburg, Russia; ^4^Institute of Fundamental Medicine and Biology, Kazan (Volga Region) Federal University, Kazan, Russia; ^5^Department of Neurology, Dagestan State Medical Academy, Makhachkala, Russia; ^6^Laboratory of Electron Microscopy, A.A. Vishnevsky Institute of Surgery, Moscow, Russia

**Keywords:** plectin, plectinopathy, limb-girdle muscle dystrophy 2Q, lung injury, muscular dystrophies

## Abstract

Plectinopathies are orphan diseases caused by *PLEC* gene mutations. *PLEC* is encoding the protein plectin, playing a role in linking cytoskeleton components in various tissues. In this study, we describe the clinical case of a 26-year-old patient with an early onset plectinopathy variant “limb-girdle muscle dystrophy type 2Q,” report histopathological and ultrastructural findings in m. vastus lateralis biopsy and a novel homozygous likely pathogenic variant (NM_201378.3:c.58G>T, NP_958780.1:p.Glu20Ter) in isoform 1f of the gene *PLEC*. The patient had an early childhood onset with retarded physical development, moderate weakness in pelvic girdle muscles, progressive weakening of limb-girdle muscles after the age of 21, pronounced atrophy of axial muscles, and hypertrophy of the gastrocnemius, deltoid, and triceps muscles, intermittent dyspnea, and no skin involvement. Findings included: non-infectious bronchiolitis and atelectasis signs, biopsy revealed myodystrophal pattern without macrophage infiltration, muscle fiber cytoskeleton disorganization resulted from the plectin loss, incomplete reparative rhabdomyogenesis, and moderate endomysial fibrosis. We have determined a novel likely pathogenic variant in PLEC 1f isoform that causes limb-girdle muscle dystrophy type 2Q and described the third case concerning an isolated myodystrophic phenotype of LGMD2Q with the likely pathogenic variant in PLEC 1f isoform. In addition, we have demonstrated the presence of severe lung injury in a patient and his siblings with the same myodystrophic phenotype and discussed the possible role of plectin deficiency in its pathogenesis.

## Introduction

Plectinopathies are a group of hereditary diseases caused by mutations in the plectin gene (*PLEC*). Clinical manifestations include skin symptoms, infantile respiratory complications, alopecia, nail and teeth dystrophic changes, laryngeal and urethral strictures, cerebral atrophy, cardiac pathology, and myasthenic and muscular-dystrophic syndromes. These phenotypic manifestations are divided into five nosological entities including limb-girdle muscular dystrophy type 2Q (LGMD2Q, OMIM #613723) (1). Four plectin isoforms (1, 1b, 1d, 1f) contribute to the complex molecular structure of the cytoskeleton and form a complex within the cell membranes of striated muscles. The most uncommon and least-studied plectinopathy, limb-girdle muscular dystrophy type 2Q, exhibits an isolated muscular-dystrophic phenotype and results from defects in isoform 1f of plectin. In this paper, we present a family case from Dagestan (Russia), in which skeletal muscle damage was associated with respiratory pathology.

All procedures were performed after patients signed a voluntary informed consent form as required by the Declaration of Helsinki (2013) and the local Ethics Committee of Dagestan State Medical Academy (Russia). All patients signed a voluntary informed consent form for publication.

## Background

**Figure 1:**
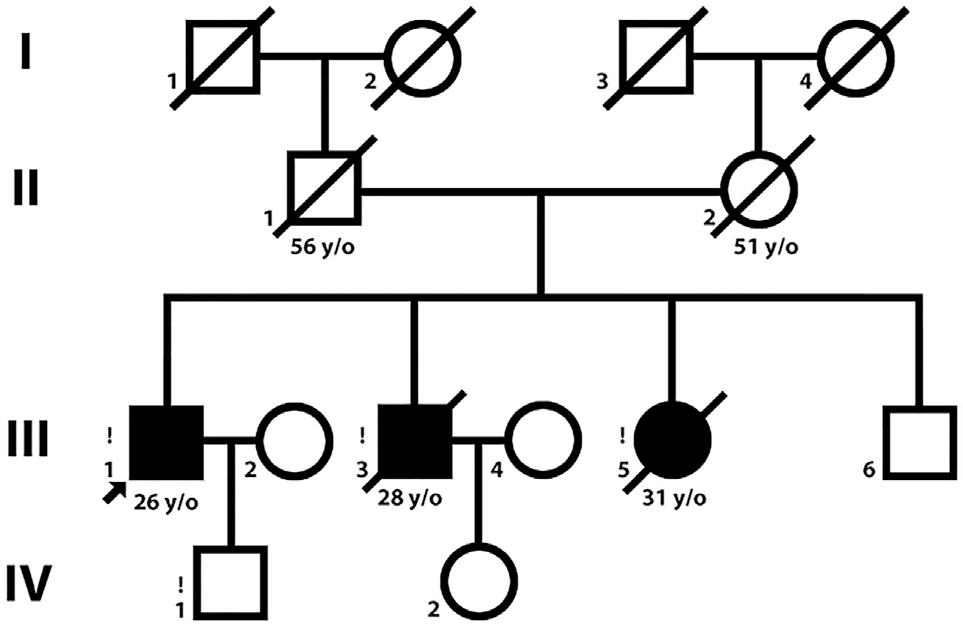
Pedigree. Symbols in black represent patients with a LGMD2Q phenotype.

Three siblings [two brothers, ages 26 and 29 (Patient 1 and Patient 2) and their sister, age 31 (Patient 3)] were examined (Figure 1). Patient 1 was examined twice, in 2014 and 2015.

Family history does not suggest any neuromuscular, dermatologic, or respiratory disorders. There were no identified intermarriages in this family. Patients 2 and 3 were specifically characterized by marked cachexia during their last year of life (body mass index = 16.7 and 16.2, respectively). The patients noticed the onset of dyspnea during slight exercise at the ages of 27–30. Patient 2 died at the age of 29 from sudden cardiac death 7 days after spontaneous pneumothorax, and Patient 3 died at the age of 31 from progressive respiratory failure.

**Figure 2:**
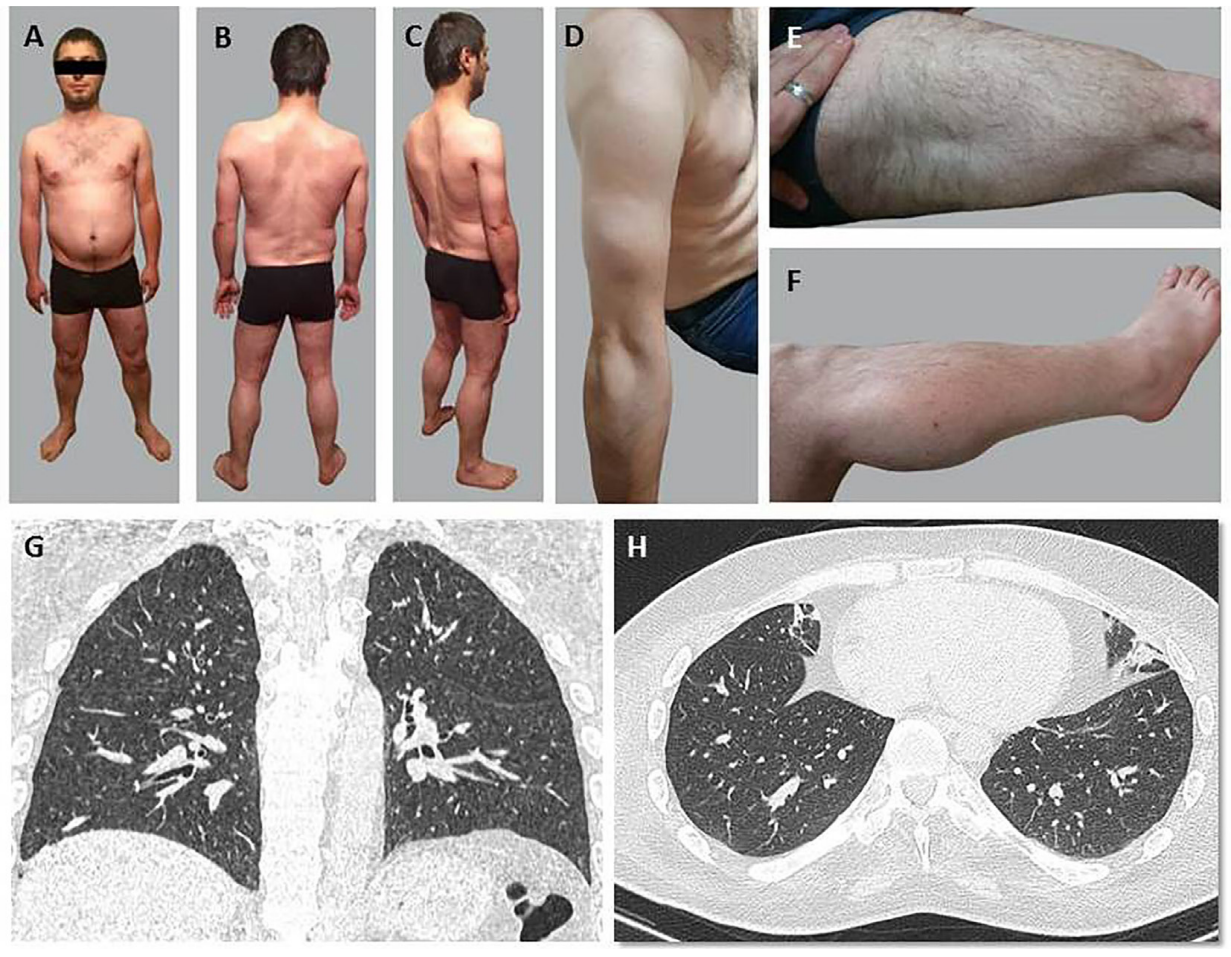
General appearance **(A–F)** and chest CT scans at the Th10-11 level **(G,H)** of Patient 1. **(A–C)** Evident amyotrophia in paravertebral muscles primarily of the thoracic spine. **(D)** Moderate amyotrophias in muscles of the shoulder girdle and proximal regions of the upper extremities. **(E,F)** Moderate pseudohypertrophy of deltoid and triceps muscles of the arm, quadriceps muscles of the thigh, and the gastrocnemius muscles. **(G,H)** A reticulo-nodular pattern in the form of slightly outlined centrilobular foci and subsegmental atelectasis in the basal areas of both lungs.

Patient 1 (Figures 2A–C) exhibited a delay in independent walking until 2.5 years old (y.o.). Muscular weakness was stable until the age of 21, afterward, progressive weakening of the muscles of the proximal regions of the lower extremities began. Since 20 y.o., he noticed progressive Achilles tendon contractures. His independent walking distance was 20–30 m with steppage occurring when tired. Episodic moderate breathlessness in everyday activities during the evening was noticed at 27 y.o.

In Patient 1, muscle weakness was more pronounced in peripheral than in proximal lower limbs (MRC scale 2/5) and more pronounced in lower than upper limbs (MRC scale 2+/5). Decreased strength, scoring 1–2 on the MRC scale, was found in mm. deltoidei, mm. erector spinae, mm. glutei, m. biceps femoris, m. adductor magnus. The results of hand strength measurement (dynamometry): *D* = 6 kgf, *L* = 4 kgf. Deep tendon reflexes were absent. Shoulder girdle and upper extremity proximal muscles exhibited moderate amyotrophy (Figure 2D), amyotrophy was also evident in the primarily thoracic spine’s paravertebral muscles. Moderate pseudohypertrophy of deltoid, triceps, quadriceps, and the gastrocnemius muscles was observed (Figures 2E,F). No abnormalities in cranial, facial, or pharyngeal muscles revealed. Tendon contractures were observed in the cervical spine.

### Laboratory Tests and Analyses

Serum CK activity level was measured in Patient 1. A standard EMG, single fiber EMG, MRI of limb and trunk muscle, ECG, EchoCG, and Chest CT scans were performed.

A high serum CK activity (3,500–4,100 U/L) was detected in Patient 1. It was also elevated in his siblings in the same range. No defects detected in the nerve conduction velocity or neuromuscular transmission by standard EMG and single fiber EMG. Myopathic motor unit action potentials were recorded. ECG changes in Patient 1 (and Patient 2) specific for an incomplete right bundle branch block, an early repolarization syndrome (and shortening of the QT interval in Patient 2 at the age of 29), combined with sudden death suggest similarity with type 2–3 Brugada syndrome. Electrophysiological signs of focal changes reflected conversion of prolonged myocardiodystrophy to focal cardiosclerosis.

Chest CT scans detected a reticulo-nodular X-ray pattern in the form of slightly outlined centrilobular foci, subsegmental atelectasis in the basal areas of both lungs and pleuropulmonary spikes in the apexes of both lungs (Figures 2G,H).

MRI of trunk muscles demonstrated marked dystrophic changes in the paravertebral muscles, primarily at the lumbar level [−70–(−60) Hounsfield units] and in a type 3 pattern (2). Changes in the mm. adductores, m. semimembranosus, m. semitendinosus, and m. biceps femoris could be characterized as highly noticeable. MRI signal intensities from m. rectus femoris and m. vastus lateralis were elevated mainly in the proximal region.

Moderate changes were seen in the anterior group of muscles and the most severe degenerative changes in the lateral group of muscles, m. soleus.

### Genetic Testing

DNA samples obtained only from Patient 1 (his siblings and parents had deceased by the time of genetic testing) were studied by whole-exome sequencing (next generation sequencing). Likely pathogenic variant was identified by comparing the patient’s genome with a normal human genome. Detected alterations were later confirmed by standard reference methods (Sanger sequencing and PCR).

**Figure 3:**
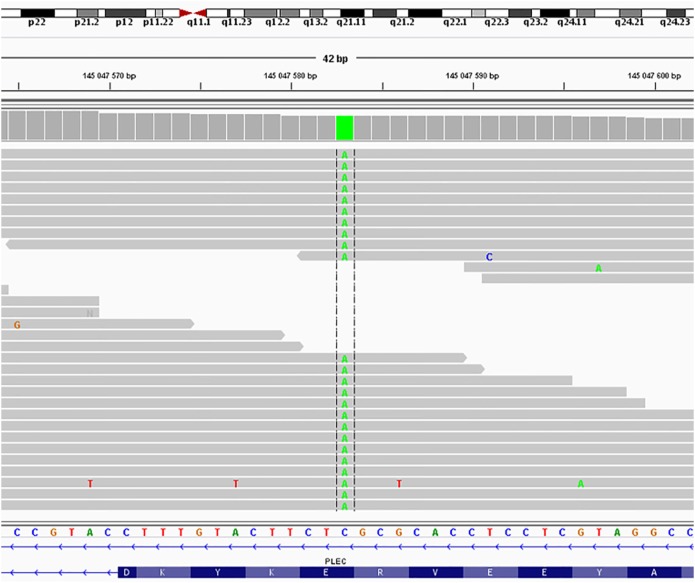
Sequence of the *PLEC*, isoform 1f from Patient 1 cDNA. The novel apparently homozygous mutation NM_201378.3:c.58G>T, NP_958780.1:p.Glu20Ter in PLEC isoform 1f was found and classified as a likely pathogenic variant according to the criteria of the American College of Medical Genetics and Genomics and the Association for Molecular Pathology (3).

New Generation Sequencing: a new apparently homozygous mutation NM_201378.3:c.58G>T, NP_958780.1:p.Glu20Ter in PLEC isoform 1f (Figure 3) was found and classified as a likely pathogenic variant according to the criteria of the American College of Medical Genetics and Genomics and the Association for Molecular Pathology (3).

### Morphological Examination

Paraffin sections of Patient 1 m. vastus lateralis biopsy samples were stained with hematoxylin, eosin, and Mallory’s trichrome, as well as immunohistologically with antibodies against plectin [rabbit antiserum #46 against rod-domains of all isoforms of plectin was a gift from Dr. Gerhard Wiche (University of Vienna, Austria)], desmin (Dako, clone D33), PCNA (Dako, clone PC10), CD34 (Leica Biosystems, clone QBEnd/10), MHCfast (Sigma Aldrich, clone MY-32), MHCslow (Sigma Aldrich, clone NOQ7.5.4D), Pax7 (marker of quiescent myosatellites) (Abcam), Myf5 (marker of activated myosatellites) (Santa Cruz, clone C-20), and myogenin (myosatellites terminal differentiation factor) (Dako, clone F5D). A gastrocnemius muscle biopsy sample of a healthy male at age 30 was used as a control. Images were acquired using a Zeiss Axio Imager.Z2 microscope with an AxioCam HRc camera (Carl Zeiss), and morphometric analysis was performed using the AxioVision Software (Carl Zeiss). Transmission electron microscopy images were acquired using a JEM-4000EX transmission electron microscope (JEOL).

**Figure 4:**
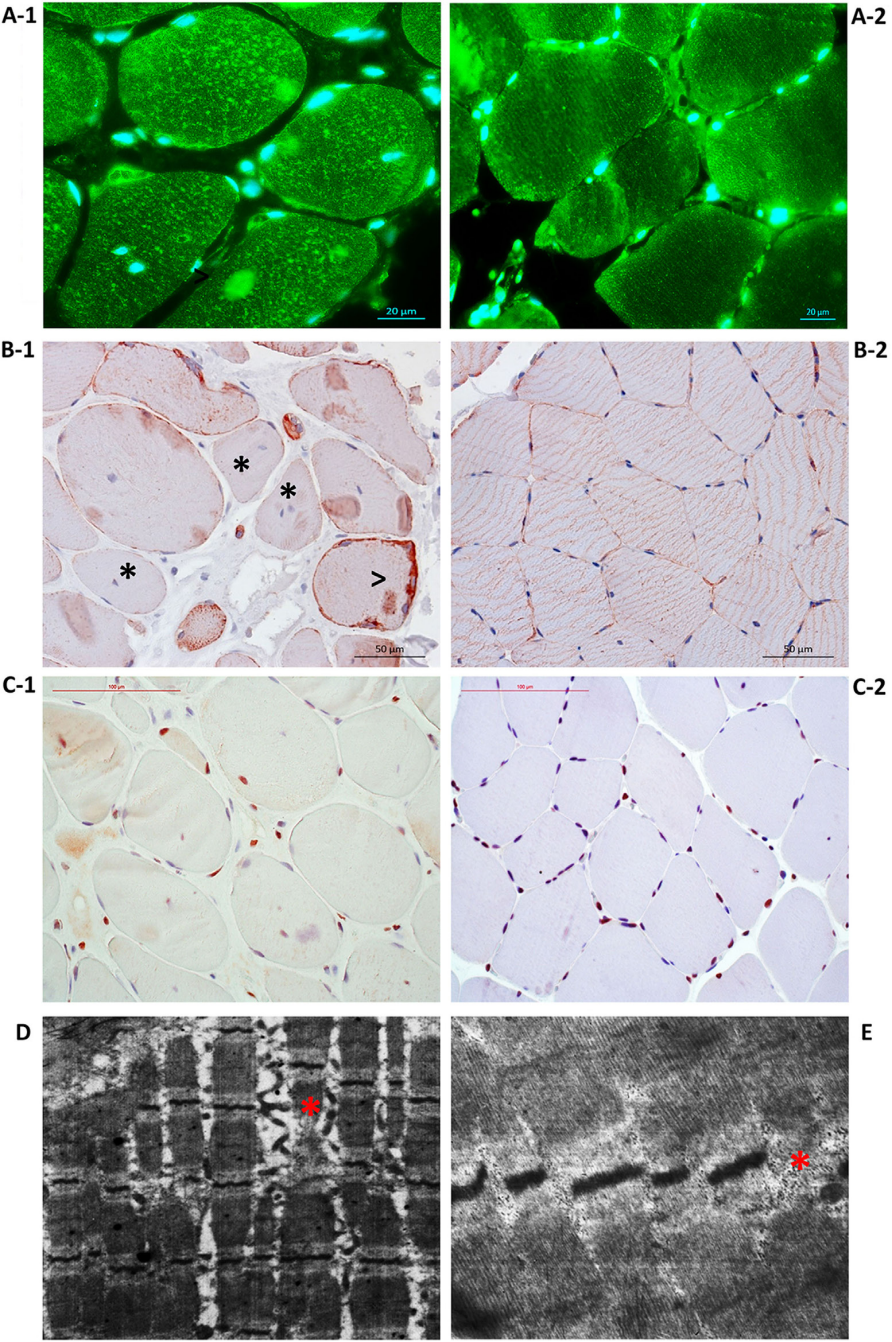
Disorganization of Patient 1 muscle fiber cytoskeleton due to the loss of plectin. **(A-1)** Immunostaining with antibodies against plectin showed plectin loss in muscle fiber sarcolemma and plectin aggregation within the sarcoplasm. **(A-2)** Control immunostaining with antibodies against plectin. **(B-1)** Immunostaining with antibodies against desmin indicated loss (asterisk) and accumulation of desmin along the periphery (arrowhead). **(B-2)** Control immunostaining with antibodies against desmin. **(C-1)** Immunostaining with antibodies against PCNA showed the tendency toward decreased proliferative activity of cells of a muscular differon and in the endomysium, enhanced proliferation of the endothelium. **(C-2)** Control immunostaining with antibodies against PCNA. Transmission electron microscopy showed dystrophic and destructive changes, Z-line disorganization [**(D)**, asterisk] and small, electron-dense mitochondria located in clusters under the sarcolemma and between sarcomeres [**(E)**, asterisk].

The primary findings of the morphometric examination of Patient 1 biopsy samples are shown in Table 1. Histopathological analysis revealed myopathic pattern with size polymorphism of muscle fibers, increased portion of central nuclear muscle fibers, moderate fibrosis, and a swollen endomysium. PCNA expression analysis demonstrated the tendency toward decreased proliferative activity of cells of a muscular differon and in the endomysium, enhanced proliferation of the endothelium (Figure 4C-1). “Number of capillaries/number of muscle fibers” ratio was closely the same, with irregular distribution of capillaries in patient’s biopsy. There was no difference in proportion of Pax7-positive nuclei and only few myogenin-positive nuclei were observed in patient’s and control biopsies, whereas proportion of Myf5-positive nuclei was significantly higher in patient’s biopsy indicating activated but unsuccessful reparative rhabdomyogenesis. There was shift toward slow myosin heavy chains expressing myofibers with fastMHC-positive myofibers of lesser diameter showing selective atrophy of fast myofibers.

**Table 1:** Morphometric characteristics of patient 1 m. vastus lateralis (mutation) vs healthy donor’s m. gastrocnemius (control). *P < 0.05.

Morphometric parameter	Mutation	Control
Muscle fiber cross-sectional area, μm^2^	3,116.26 ± 2,312.63	3,580.99 ± 801.25
Portion of central nuclear muscle fibers, %	**39.37 ± 7.76**	1.37 ± 0.15*
Relative area of connective tissue, %	**17.58 ± 0.01**	1.64 ± 0.38*
Portion of fibers expressing fast/slow myosin heavy chains, %	52.03/47.97	67.3/37.8 (Johnson et al., 1973)
Proportion of Pax7-positive nuclei, %	48.66 ± 4.15	57.33 ± 7.05
Proportion of Myf5-positive nuclei, %	**37.64 ± 10.24**	2.27 ± 1.79*
“Number of capillaries/number of muscle fibers” ratio	2.90 ± 0.07	2.96 ± 0.86

*Numbers in bold are statistically significant values*.

Immunofluorescence imaging of plectin in the biopsy samples revealed a loss of plectin in muscle fiber sarcolemma and plectin aggregates with irregular size and shape and fuzzy margins (Figure 4A-1). Desmin was distributed unevenly within the muscle fibers of Patient 1 biopsy samples with accumulation along the periphery; these results suggest disorganization of the muscle fiber cytoskeleton due to the loss of plectin (Figure 4B-1).

Electron microscopy revealed dystrophic and destructive changes in the biopsy samples. *Z*-line disorganization was identified, as well as thinned myofibrils with enlarged spaces between them (Figure 4D). The presence of foci of myofibril destruction resulted in spaces with membrane fragments and a flaked content. Up to 30 mitochondria were observed and found to be small, electron-dense, and located in clusters under the sarcolemma and between sarcomeres (Figure 4E). In some areas, myofibrils underwent fragmentation and disorganization, the myofibril disruptions noticed within the disks I. Evidence of damaged sarcomeres were also observed.

### Statistical Analysis

The results of the morphometric analysis are presented as the mean ± SD. Significant changes were assessed by Student’s *t*-test, with *P* < 0.05 as the level of significance.

## Discussion

Only two reports have previously been published concerning an isolated myodystrophic phenotype of limb-girdle muscle dystrophy 2Q caused by a mutation in plectin isoform 1f. In 2010, Gundesli et al. described six patients from three independent closely related Turkish families with limb-girdle muscle dystrophy 2Q (4). This report identified a homozygous deletion (1_9delATGGCCGGC) in the gene *PLEC* within the 1f exon and including the start codon.

In 2015, Fattahi et al. reported a plectinopathy in an isolated consanguineous Iranian family (two affected sisters). The dynamics and character of the clinical manifestations were similar to those of our case except for concomitant symptoms such as ptosis, ophthalmoparesis, and dysphagia, which were considered to be manifestations of a myasthenic syndrome (5). Whole-exome sequencing detected heterozygous compound mutations p.Gln1022Ter (c.3064C>T) and p.Gly3835Ser (c.11503G>A) localized in the N- and C-terminal globular domains of plectin 1f. In this case, mutation in the C-terminal domain was suggested to lead to the development of a myasthenic syndrome. This claim is supported by genetic analysis of other plectinopathy cases associated with a myasthenic syndrome wherein mutations in the C-terminal domain (an interval of 10187–12043 bp in exon 32), a universal globular domain contained in all plectin isoforms, were also identified (6).

Plectin isoform 1f has been recently shown in a model of plectin-deficient mice to be required to maintain the continuity of a neuromuscular synapse when it functions as a linker between acetylcholine receptors and intermediate filaments of the cytoplasm *via* rapsyn (7). However, in our patient, as in the case of Gundesli et al., no manifestations of a myasthenic syndrome as seen in other plectinopathies were observed to implicate plectin isoform 1f in stabilization of the human neuromuscular junction. The clinical picture of our patient was similar to the phenotypes previously described for mutations in PLEC isoform 1f distinctive for the presence of pulmonary damage, which clinically manifested as breathlessness during evening activities. His siblings also suffered from dyspnea from the age of 27–30 years that subsequently resulted in their deaths from spontaneous pneumothorax and respiratory failure, which suggests a higher pathogenicity of this mutation than that previously described by Gundesli et al. and Fattahi et al. To the best of our knowledge, the role of plectin isoform 1f in the development of pulmonary diseases has not been studied to date. However, it is known that this isoform is involved in transmitting mechanoreceptor stimuli from the extracellular matrix *via* dystroglycan to intracellular cascades of kinases ERK1/2 (extracellular signal-regulated kinases 1/2) and AMPK (AMP-activated protein kinase) in alveolocytes (8). It has been shown that activation of AMPK and ERK1/2 can be cytoprotective (9, 10) and that insufficient activation of these pathways resulting from mutations in plectin isoform 1f can result in alveolocyte damage and trigger an inflammatory reaction. It is also possible that alveolocyte damage results from the destruction of focal adherens junctions that contain plectin and connect the intercellular matrix and the cytoskeleton Eisenberg et al. (11). At present, the involvement of lungs in plectinopathies has only been described for cases of epidermolysis bullosa as damage to the mucous membranes of the trachea and bronchi (12).

## Concluding Remarks

We have determined a novel likely pathogenic variant in PLEC 1f isoform that causes limb-girdle muscle dystrophy type 2Q and described the third case with an isolated myodystrophic phenotype of LGMD2Q with the in PLEC 1f isoform. In addition, we have demonstrated the presence of severe lung injury in a patient and his siblings with the same myodystrophic phenotype and discussed the possible role of plectin deficiency in its pathogenesis.

## Ethics Statement

All procedures were performed after patients signed a voluntary informed consent form as required by the Declaration of Helsinki (2013) and the local Ethics Committee of Dagestan State Medical Academy (Russia). All patients signed a voluntary informed consent form for publication.

## Author Contributions

Collecting the data: RD, SB, ZU, PA, RM, IC, and GD. Analyzing the data: RD, SB, MM, IY, and AI. Interpreting the data: RD, SB, MM, IY, and AI. Drafting the manuscript: RD, SB, MM, and IY. Morphological examination: RD and MM. Genetic testing: IC, GD, and AI. The concept of this research: RD.

## Conflict of Interest Statement

The authors declare that the research was conducted in the absence of any commercial or financial relationships that could be construed as a potential conflict of interest.
